# Effects of long-term antibiotic treatment on mice urinary aromatic amino acid profiles

**DOI:** 10.1042/BSR20203498

**Published:** 2021-01-05

**Authors:** Xuehang Zhu, Bin Fu, Manyuan Dong, Yangyang Guo, Zheng Cao, Junfang Wu

**Affiliations:** 1 State Key Laboratory of Magnetic Resonance and Atomic and Molecular Physics, Key Laboratory of Magnetic Resonance in Biological Systems, Wuhan Center for Magnetic Resonance, Wuhan Institute of Physics and Mathematics, Innovation Academy for Precision Measurement Science and Technology, Chinese Academy of Sciences, Wuhan, China; 2 University of Chinese Academy of Sciences, Beijing, China; 3 Hubei Provincial Hospital of Integrated Chinese and Western Medicine, Hubei University of Chinese Medicine, Wuhan, China; 4 Division of Cardiology, Department of Internal Medicine, Tongji Hospital, Tongji Medical College, Huazhong University of Science and Technology, Wuhan, China

**Keywords:** aromatic amino acids, dysbiosis, host-microbial co-metabolites, long-term antibiotics treatment, microbiome

## Abstract

The gut microbiota–host co-metabolites are good indicators for representing the cross-talk between host and gut microbiota in a bi-direct manner. There is increasing evidence that levels of aromatic amino acids (AAAs) are associated with the alteration of intestinal microbial community though the effects of long-term microbial disturbance remain unclear. Here we monitored the gut microbiota composition and host–microbiota co-metabolites AAA profiles of mice after gentamicin and ceftriaxone treatments for nearly 4 months since their weaning to reveal the relationship between host and microbiome in long- term microbial disturbances. The study was performed employing targeted LC-MS measurement of AAA-related metabolites and 16S RNA sequence of mice cecal contents. The results showed obvious decreased gut microbial diversity and decreased *Firmicutes/Bacteroidetes* ratio in the cecal contents after long-term antibiotics treatment. The accumulated AAA (tyrosine, phenylalanine and tryptophan) and re-distribution of their downstreaming metabolites that produced under the existence of intestinal flora were found in mice treated with antibiotics for 4 months. Our results suggested that the long-term antibiotic treatment significantly changed the composition of the gut microbiota and destroyed the homeostasis in the intestinal metabolism. And the urinary AAA could be an indicator for exploring interactions between host and gut microbiota.

## Introduction

There are numerous gut microbes that exist in the digestive tract, which constitute a complex ecosystem [1]. The cross-talk between host and gut microbiota has been well established in a bi-direct manner. That is, symbiotic microbes rely on the nutrient-rich environment provided by the gut. On the other hand, gut microbes play an important role in regulating the host’s physiological functions, including processing nutrient substances, maintaining energy balance [2], and improving the immune system [3]. Studies have found that the disruption of the microbial ecological balance or the loss of bacterial diversity would lead to pathophysiological processes in the host. For example, lack of gut microbes increased the incidence of hyperglycemia, adiposity, and insulin resistance in the germ-free mice model [4]. In turn, regulation of the microbial flora could modulate the gastrointestinal physiology, promote intestinal inflammation, and alleviate the non-alcohol fatty liver disease or neuropsychiatric diseases [5,6]. But the effects of long-term microbial disturbance on host remain unclear.

Previous studies have shown the metabolites are good indicators for representing the cross-talk between the host and the gut microbiota. Gut microbiota can regulate the gut peptides (such as serotonin (5-HT) and dopamine (DA)) through neuroendocrine or the vagal afferent pathway via the microbiota–gut–brain axis [7]. Those metabolites produced under the existence of microbiota, such as bile acids [8], short-chain fatty acids, and trimethyl-*N*-oxide can play physiological roles in the host [6,9]. Besides, the aromatic amino acids (AAAs), including phenylalanine, tryptophan, and tyrosine, are kind of essential amino acids that participate in the cross-talk between host and its microbiota. Some of the AAAs, which mainly come from dietary food, undergo digestion in the intestine, and the rest escape intestinal digestion could serve as fermentable substrates for further metabolism by gut microbiota [10]. The increased AAA is a risk that associated with insulin resistance and diabetes [11]. Dodd et al. found *Clostridium sporogenes* could modulate serum AAA levels and affect intestinal permeability and systemic immunity in a gnotobiotic mice model [12]. Taken together, there is accumulating evidence that levels of AAA are associated with the disturbance of the intestinal microbial community.

In this work, two representative antibiotics, gentamicin and ceftriaxone, were selected to establish a mice model for observing the effects of long-term antibiotics on host and its intestinal flora metabolism. Of them, gentamicin belongs to aminoglycosides and its antibacterial spectrum is mainly Gram-negative bacteria. It can combine with bacterial 30S ribosome subunits for blocking the synthesis of bacterial proteins. Ceftriaxone is a β-lactam antibiotic that inhibit cell wall formation and facilitate its cracking. It has a strong antibacterial effect on both Gram-negative and -positive bacteria. The reasons for choosing these two antibiotics are: first, these two antibiotics are not absorbable by the host following oral administration [13], which are suitable for establishing a mice model with local intestinal flora disturbance and investigating the metabolic changes on local microbial dysbiosis. Second, both of them are broad-spectrum antibiotics that can widely inhibit intestinal flora in a certain complementary way to investigate the effects of combined antibiotics on host intestinal flora. Third, these two antibiotics metabolize very quickly by oral administration so that it has less impact on other organs except for intestinal tracts to avoid antibiotic toxicity (such as gentamicin-induced renal toxicity and hearing loss) at the greatest extent. Our previous works have found a 4-day short-term combined gentamicin and ceftriaxone treatment suppressed bacterial fermentation and stimulated protein degradation [14]. However, the long-term antibiotic effects on metabolic profiling remain unknown.

Here we established a 4-month continuous long-term antibiotic treated mice model which started from 3 weeks since weaning. To reveal the relationship between the host and its gut microbes, the urinary AAA metabolites and cecal gut microbial profiling were measured by a combination of targeted liquid chromatography-tandem mass spectrometry (LC-MS/MS) and 16S ribosomal RNA gene sequencing. The interactions between changed AAA and gut microbiota were calculated to illustrate their correlation.

## Materials and methods

### Animal experiments and sample collection

Animals in experiments were bred by mating male and female C57BL/6 mice in SPF animal facility under standard conditions (temperature, ∼20°C; humidity, 40−70%; 12-h/12-h light/dark cycle) with free access to chow diet and water at Wuhan Institute of Physics and Mathematics (SYXK 2015-0051). The F1 hybrid male and female C57BL/6 mice were purchased from Hunan Slyk Jingda Experimental Animals Co., Ltd. The experiment started with mice aged 3 weeks at their weaning and received the same standard diet according to laboratory animals’ nutrients for formula feeds of China (GB14924.3-2010) all through the experiment. The mice were divided into two groups (Group C: control, *n*=8, Group A: control mice treated with antibiotics, *n*=8). The mice in group A received combined antibiotics at low doses by oral gavage for consecutive 13 weeks (sulfate gentamicin: 180 mg/kg/day; ceftriaxone sodium: 500 mg/kg/day). Mice in the control group were treated with equal vehicle (0.9% saline) once a day until killing at the 16th week. All experiments and procedures were adhering to the National Guidelines for Experimental Animal Welfare and the ethics approval were confirmed by the research ethics committee at Wuhan Institute of Physics and Mathematics, Chinese Academy of Sciences.

The urine samples were collected 1 day before killing at week 16. Mice were killed by cervical dislocation under isoflurane anesthesia to obtain serum. The colon was quickly cut out for freezing in liquid nitrogen. Cecal contents were gently squeezed out of the excised cecum into the Eppendorf tube and flash-frozen. All samples were stored at −80°C until further analysis.

### 16S ribosomal RNA gene sequencing

Microbial DNA was extracted from the cecal content of the mice, and the bacteria 16S ribosomal RNA gene amplicon sequence library was prepared. The equimolar amounts of purified DNA amplicons were further amplified on an Illumina MiSeq platform for paired-end sequencing (2× 300 bp) according to the standard protocols by Shanghai Major Bio-Pharm Technology Co., Ltd. The raw fastq files were then demultiplexed and quality-filtered (Phred quality score ≥ 20) using QIIME (version 1.9.1). The Operational Taxonomic Units (OTUs) table was clustered with a 97% similarity cutoff based on the open-reference approach using UCLUST.

### Targeted metabolite quantification by LC-MS/MS

Targeted host–microbiota related co-metabolites in urine were measured on Agilent 6490 triple quadrupole LC-MS/MS equipped with electrospray ion source. Briefly, The internal standard (IS) mixture was prepared with benzoic acid-C_13_ (200 μM), homovanillic acid-d_5_ (200 μM), 4-hydroxybenzoic acid-C_13_ (25 μM), 4-hydroxy-3-methoxymandelic acid-d_3_ (25 μM), indole-3-acetic acid-d_2_ (10 μM), nicotinamide-d_4_ (1.25 μM), 3-hydroxytryptamine-d_2_ (5 μM), l-phenylalanine-N_15_ (50 μM), l-tyrosine-C_13_ (25 μM), hippuric acid-N_15_ (400 μM), and l-tryptophan-d_5_ (100 μM). Then 20 μl of IS mixture and 170 μl of deionized water was added into 10 μl of the urine sample. After vortex and centrifugation (12000 rpm, 4°C, 10 min), the supernatant was filtered by 0.22-μM filter membrane before LC-MS/MS measurement. The metabolites were quantified with the ISs. At the same time, the creatinine concentration was tested to be a reference in the quantification of host–microbiota co-metabolites in urine samples. The concentration of urinary co-metabolites was expressed as μmol per liter urine relative to mmol/l of creatinine.

To further investigate the tryptophan pathway-related metabolites in serum and cecal contents, 10 μl of serum was mixed with 10 μl of IS (trp-d_5_, 2 μg/ml) and 10 μl of 50% acetonitrile (ACN) containing 0.1% formic acid. Subsequently, 150 μl ice-cold methanol was added into this mixture and stood for 30 min at −20°C for protein precipitation. After centrifugation for 15 min (3000×***g***, 4°C), the supernatants were collected and evaporated to dryness with a concentrator [15]. The dried extracts were reconstituted in 100 μl of 50% ACN containing 0.1% formic acid. Two microliter was injected into the LC-MS system. For sample preparation in cecal contents, ∼10 mg samples were mixed with 400 μl ice-cold methanol, 50 μl of 50% ACN containing 0.1% formic acid, and 10 μl of IS (trp-d_5_, 2 μg/ml). Then the mixture was homogenized by tissuelyzer for 90 s at 25 Hz. After centrifugation and evaporation [15], the dried extracts were reconstituted in 200 μl of 50% ACN containing 0.1% formic acid. Quantification of tryptophan metabolites was performed using calibration curves and ratios of the integrated peak areas of their ISs.

### RNA extraction and RT-qPCR of colon tissue

Total RNA was extracted from colon tissue using RNAiso Plus (TAKARA, JAPAN) according to the manufacturer’s instructions. Then 1 μg RNA was extracted for reverse transcription synthesis reaction. The complementary DNA (cDNA) was synthesized with transcript all-in-one first-strand cDNA synthesis supermix for qPCR (Transgene, Beijing). The cDNA was diluted with 1:10, and then 1 μl was used for each qPCR. The qPCR was performed using SYBR Green reagent (Applied Biosystems, CA, U.S.A.) on a Step-One Real-Time PCR System (Applied Biosystems) with the following specific gene primers were prepared for Aryl hydrocarbon receptor (AHR): Forward Prime 5′-TCGGGGTACC AGTTCATCCA-3′, Reverse Prime 5′-ACCTCCAGCGACTGTGTTTT-3′. The qPCR conditions were set to 40 cycles of 95°C for 20 s, 95°C for 30 s, and 60°C for 30 s. The GAPDH was chosen as a housekeeping gene for each experiment. The relative expression was calculated using the formula 2^−ΔΔ*C*_t_^ for analysis.

### Statistical analysis

Statistical analyses and data mapping were performed using SPSS 25 (SPSS Inc., Chicago, IL, U.S.A.) and GraphPad Prism 8 statistical software (La Jolla, CA, U.S.A.). Independent Student’s *t* test (two-tailed) was performed for comparison between two groups. Otherwise, the non-parametric Mann–Whitney U test was used for those data that did not pass the homogeneity of variance test. The *P*<0.05 with FDR correction was considered statistically significant. All values were expressed as Mean ± S.D. The statistical analysis about 16S ribosomal RNA gene sequencing was performed on the I-Sanger cloud platform, and data visualization was conducted in the R environment.

## Results

### Effects of long-term antibiotics treatment on mice bodyweight and gut microbial profiling

**Figure 1:**
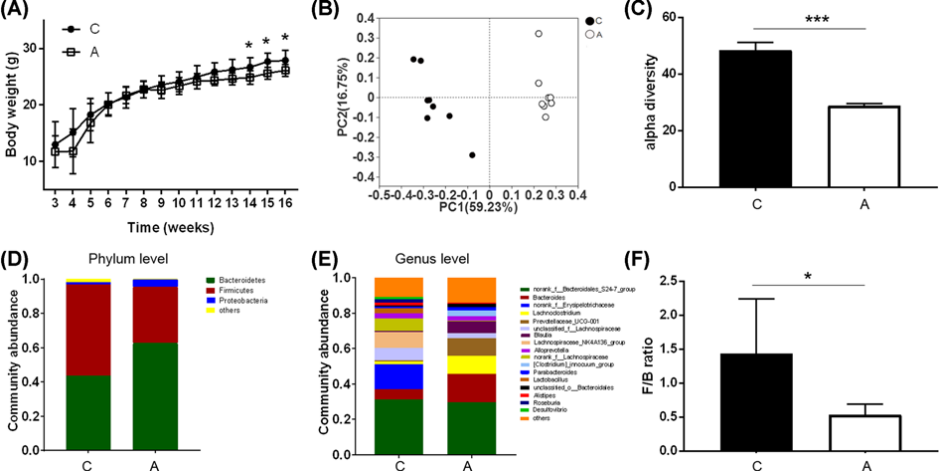
Effects of long-term antibiotics treatment on mice bodyweight and gut microbial profiling (**A**) Bodyweight changes of mice treated with antibiotics and its equal vehicle in 16 weeks. (**B**) The PCoA plot for the compared two groups on genus level. (**C**) The α diversity in the gut microbiome of mice treated with vehicle and antibiotics (*P*=0.0007). (**D,E**) Community abundance bar plot analysis in the gut microbiome of mice treated with vehicle and antibiotics on phylum (D) and genus (E) levels. (**F**) The F/B ratio decreased in mice treated with antibiotics compared with those treated with vehicle (*P*=0.0148). Keys: C, control mice; A, control mice treated with antibiotics. Data are presented as mean ± SD; *n*=8 per group; *P<0.05, ***P<0.001 by two-tailed Student’s *t* test or Mann−Whitney test. Abbreviation: PCoA, principal coordinated analysis.

Compared with the control group, the body weight of mice treated with long-term antibiotics did not show significant differences at an early stage. Since 11 weeks after antibiotics treatment, bodyweight in group A mice started to display a significant decrease compared with control ones (Figure 1A).

To investigate the effects of long-term antibiotics treatment on microbial profiling, the 16S gene sequence in mice cecal contents were measured in both groups. The gut microbiota richness was measured by numbers of observed OTU. The principal coordinated analysis (PCoA) based on the Bray–Curtis distance algorithm revealed a clear cluster for mice treated with antibiotics and the control ones (Figure 1B). From the phylogenetic diversity index, mice in group A showed significant down-regulation of α diversity, indicating that the suppressed gut microbiota diversity following by antibiotics treatment (Figure 1C). The relative abundance of taxa was assessed at different levels of both groups to investigate the specific changes of the gut microbiome. At the phylum level, the *Bacteroidetes* and *Firmicutes* are the dominant bacteria in the gut microbiome for both the groups. The increased abundance of *Bacteroidetes* and *Proteobacteria*, together with decreased abundance of *Firmicutes*, were changed significantly in mice treated with antibiotics from the relative percent community abundance (Figure 1D). The *Firmicutes/Bacteroidetes* ratio was decreased significantly in the antibiotics-treated group compared with the control ones (Figure 1F). At the genus level, the community bar plot presented an increased abundance of *Bacteroides, Lachnoclostridium, Prevotellaceae, Blautia*, and a decreased abundance of *Erysipelotrichaceae* in group A (Figure 1E). These results indicated the disturbed gut microbiome profiling induced by the long-term antibiotic intervention.

### Effects of long-term antibiotics on metabolic profiling by targeted LC-MS measurement

The AAA and their downstream metabolites produced with the existence of gut flora in urine were quantified by targeted LC-MS measurements to explore the interaction between the host and its gut microbiota.

**Figure 2:**
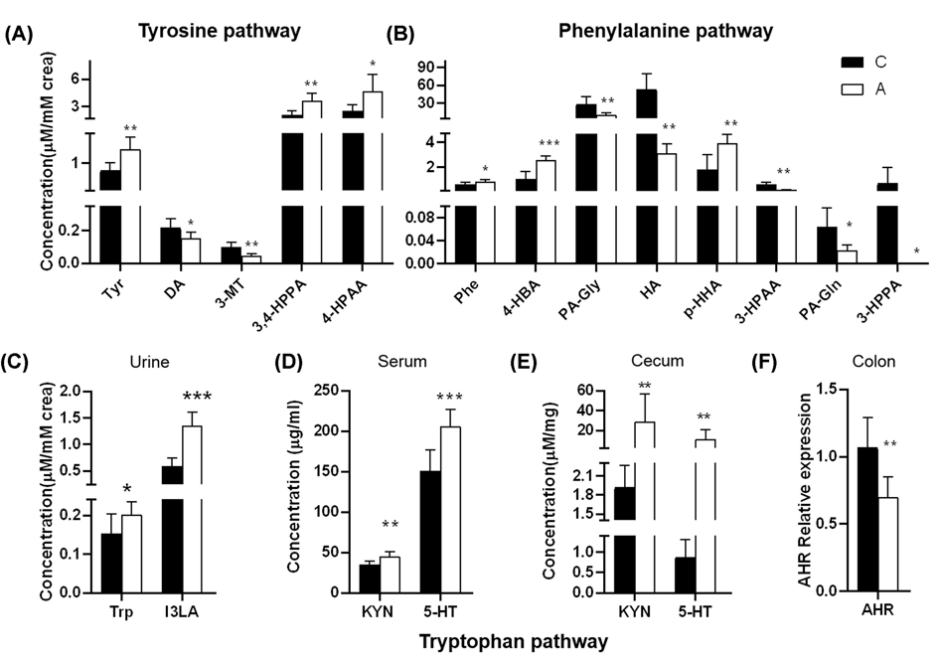
Quantification of three kinds of AAAs co-metabolism metabolites in mice treated with vehicle and antibiotics Quantification of changed urinary metabolites involved in (**A**) tyrosine metabolism, (**B**) phenylalanine metabolism, and (**C**) tryptophan metabolism. (**D,E**) Quantification of tryptophan-related metabolites in serum sample (**D**) and cecum content (**E**) of mice treated with vehicle and antibiotics. (**F**) AHR mRNA expression in the colon of mice treated with vehicle and antibiotics. Keys: C, control mice; A, control mice treated with antibiotics; PAA, phenylacetic acid; Phe, phenylalanine; Trp, tryptophan; Tyr, tyrosine. Data are presented as mean ± SD; *n*=8 per group; *P<0.05, **P<0.01, and ***P<0.001, by two-tailed Student’s *t* test or Mann−Whitney test.

Of the 26 quantified urinary metabolites, 15 were changed significantly in mice treated with long-term antibiotics compared with the controls. There were five changed metabolites related to tyrosine metabolism (Figure 2A and Table 1). The levels of tyrosine, 3-(3,4-dihydroxy phenyl) propionic acid (3,4-HPPA), and 4-hydroxyphenyl acetic acid (4-HPAA) were increased in group A whereas the levels of DA and 3-methoxytyramine (3-MT) were decreased significantly. There were eight changed metabolites involved in phenylalanine metabolism. The increased levels of urinary phenylalanine, 4-hydroxybenzoic acid (4-HBA), and *p*-hydroxyhippuric acid (*p*-HHA), whereas the decreased levels of phenylacetyl glycine (PA-Gly), hippuric acid (HA), 3-hydroxyphenyl acetic acid (3-HPAA), phenylacetyl glutamine (PA-Gln), and 3-(3-hydroxyphenyl) propanoic acid (3-HPPA) were found in antibiotics-treated mice (Figure 2B and Table 1). The levels of tryptophan and indole-3-lactic acid (I3LA) in tryptophan metabolism were increased significantly in group A (Figure 2C and Table 1). Such changes implied the accumulated AAA and the re-distribution of the host–microbiota co-metabolites following antibiotic treatment.

**Table 1:** The concentration of measured urinary host–microbial AAA co-metabolites in two groups

Concentration (μM)	C	A
Tyrosine pathway		
Tyr	0.83 ± 0.19	1.33 ± 0.29
DA	0.22 ± 0.06	0.15 ± 0.04
3-MT	0.1 ± 0.03	0.05 ± 0.01
3,4-HPPA	2.06 ± 0.47	3.61 ± 0.86
4-HPAA	2.55 ± 0.69	4.67 ± 1.86
Phenylalanine pathway		
Phe	0.6 ± 0.22	0.83 ± 0.18
*p*-HHA	1.82 ± 1.21	3.92 ± 0.71
4-HBA	1.07 ± 0.61	2.58 ± 0.33
HA	53.31 ± 25.52	3.12 ± 0.79
PA-Gln	0.06 ± 0.03	0.02 ± 0.01
PA-Gly	29.05 ± 11.89	10.89 ± 3.15
3-HPAA	0.67 ± 0.15	0.22 ± 0.03
2-HHA	0.18 ± 0.08	0.17 ± 0.04
3-HPPA	0.74 ± 1.29	N.D.
PAA	0.31 ± 0.17	0.14 ± 0.09
2-HPAA	0.4 ± 0.13	0.33 ± 0.14
Tryptophan pathway		
Trp	0.15 ± 0.05	0.2 ± 0.03
XAN	1.13 ± 1.02	1.07 ± 1.26
Tryptamine	0.04 ± 0.01	0.04 ± 0.01
KYN	0.59 ± 0.3	0.69 ± 0.29
Tryptophanol	0.005 ± 0.003	N.D.
5-HIAA	1.13 ± 0.25	1.24 ± 0.19
5-HT	0.08 ± 0.02	0.08 ± 0.01
I3LA	0.59 ± 0.16	1.36 ± 0.26
I3AA	0.08 ± 0.03	0.05 ± 0.03
NAM	2.87 ± 1.13	2.18 ± 1.67

All values were presented as mean ± SD. Abbreviations: C, control; A, control mice treated with antibiotics; I3AA, indole-3-acetic acid; KYN, kynurenine; NAM, nicotinamide; ND, not detected; PAA, phenylacetic acid; Phe, phenylalanine; Trp, Tryptophan; Tyr, Tyrosine; XAN, xanthurenic acid; 2-HHA, 2-hydroxyhippuric acid; 2-HPAA, 2-hydroxyphenylacetic acid; 5-HIAA, 5-hydroxyindole-3-acetic acid.

To further investigate three pathways involving in tryptophan metabolism, a total of seven metabolites were measured by targeted LC-MS/MS in serum and cecal contents in both groups (Table 2). The metabolites in serum showed a similar pattern with those in cecal contents. The levels of kynurenine (KYN) and serotonin in serum, as well as the cecal content of mice treated with antibiotics, were significantly increased (Figure 2D,E). Even we did not find significant changes in indole; however, its receptor, the AHR mRNA expression declined significantly in colon tissue from antibiotics-treated mice (Figure 2F), which indicated stimulated conversion from tryptophan into KYN and 5-HT pathway under the suppressed intestinal micro-ecology following long-term antibiotics treatment.

**Table 2:** The concentration of measured tryptophan-related metabolites in two groups

Concentration (μM)	C	A
Metabolites in serum		
Indole	3.83 ± 0.48	4.42 ± 1.13
KA	4.97 ± 0.72	5.22 ± 0.67
KYN	34.82 ± 4.23	44.82 ± 5.83
PA	4.95 ± 0.8	5.23 ± 1.78
5-HT	150.86 ± 26.23	206.44 ± 20.83
Trp	1635.29 ± 306.2	1881.65 ± 272.59
XAN	12.86 ± 0.93	33.71 ± 25.6
Metabolites in cecum		
Indole	0.21 ± 0.05	0.3 ± 0.14
KA	0.46 ± 0.13	0.95 ± 1.39
KYN	1.92 ± 0.34	29.02 ± 27.84
PA	4.16 ± 4.37	2.86 ± 2.37
5-HT	0.88 ± 0.43	11.07 ± 9.92
Trp	35.96 ± 9.98	41.55 ± 35.94
XAN	1.86 ± 0.54	3.44 ± 4.28

All values were presented as mean ± SD. Abbreviations: C, control; A, control mice treated with antibiotics; KA, kynurenic acid; PA, picolinic acid; Trp, tryptophan; XAN, xanthurenic acid.

### Correlation between the gut microbiome and metabolic profiles

**Figure 3:**
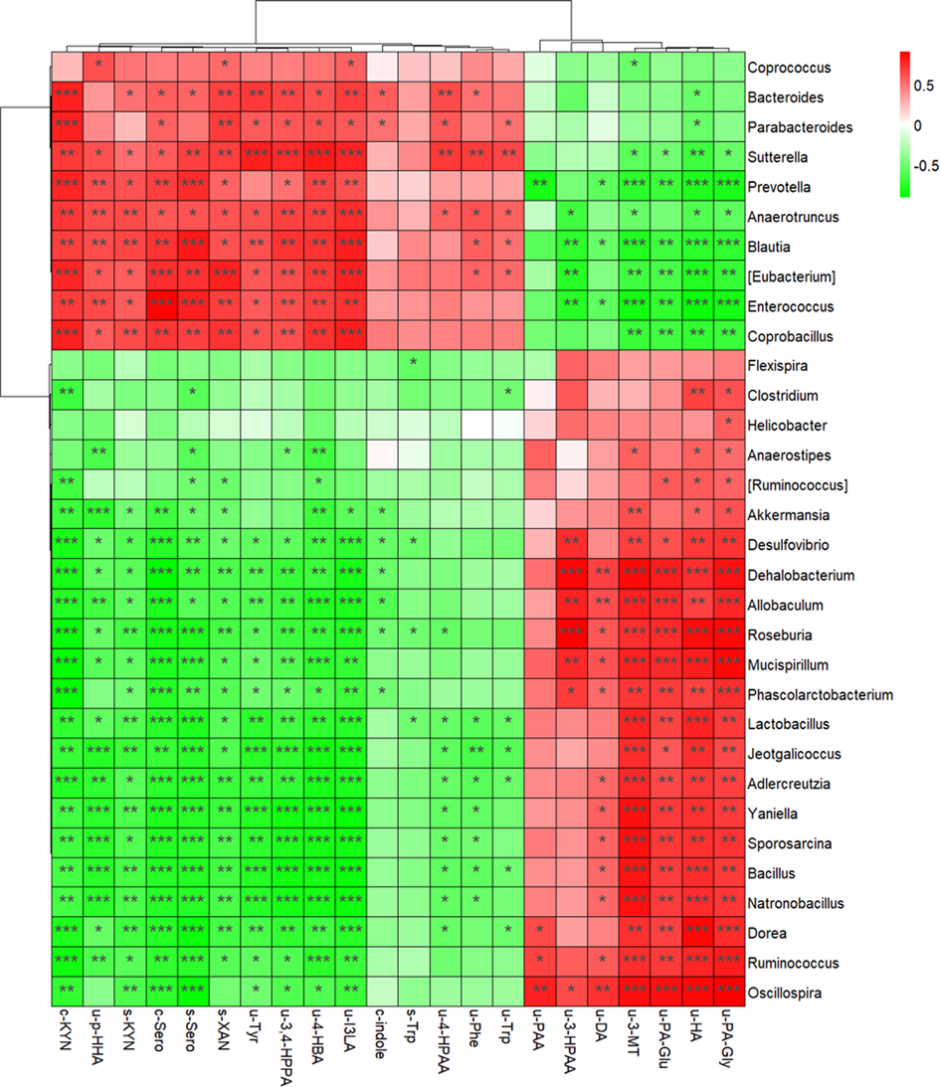
Correlations between gut microbiota on genus level and metabolites involved in the AAA pathway The correlation was calculated by the Spearman correlation. *P<0.05, **P<0.01 and ***P<0.001. Abbreviations: C, Cecal content; I3AA, indole-3-acetic acid; PAA, phenylacetic acid; S, serum; Trp, tryptophan; Phe, phenylalanine; Tyr, tyrosine; U, urine.

The connection between changed gut microbiota and AAA was presented in a heat map to present the correlation between bacterial species and AAA metabolites in measured biofluid samples of antibiotic-treated mice (Figure 3). The increased abundance of *Bacteroides* showed a highly positive correlation with increased AAA and their downstreaming products 4-HPAA, I3LA, 4-HBA, and 3,4-HPPA. Except for the above positive correlation, the *Provotellaceae, Blautia*, and *Lachnoclostridium* that belong to the *Bacteroidetes* phylum also showed a negative correlation with 3-MT and DA which are involved in the tyrosine pathway, as well as a negative correlation with PA-Gln, PA-Gly, HA, and 3-HPPA that are involved in the phenylalanine pathway. The decreased abundance of several genera (such as *Lachnospiraceae* and *Erysipelotrichacea*) belong to the *Firmicutes* phylum showed a highly positive correlation with PA-Gln, PA-Gly, HA, and 3-HPPA involved in the phenylalanine pathway, together with a negative correlation with 4-HBA in the phenylalanine pathway, 3,4-HPPA in the tyrosine pathway, and KYN, XAN (Xanthurenic acid), I3LA that are involved in the tryptophan pathway.

## Discussion

The gut microbiome is known to play a vital role in human health. To investigate the metabolic relationship between host and microbiome in long-term gut microbiota disturbance, we investigated the gut microbial composition and urinary AAA profiles of mice following combined gentamicin and ceftriaxone treatments after weaning for nearly 4 months by integrating metagenomics and metabolomics. Our study showed that long-term antibiotic treatment induced the re-distribution of the gut microbiome and unbalanced host–microbiota co-metabolism of AAA, which provide direct evidence for the gut microbiota that participates in AAA metabolism.

The F/B ratio comes from the most abundant phyla (*Firmicutes* and *Bacteroidetes*) in the gut microbiome, is associated with several pathological conditions. The increased F/B ratio has been found in non-alcoholic fatty liver related to obese youth due to a lower abundance of *Bacteroidetes, Prevotella, Gemmiger* in phylum *Bacteroidetes* [16]. Ley et al. bring out the point that the F/B ratio could be a very first index derived from the dysbiotic microbiota of obesity [17,18]. The imbalances in the F/B ratio implied the disturbed microbial diversity or abundance in the antibiotics treated mice model.

**Figure 4:**
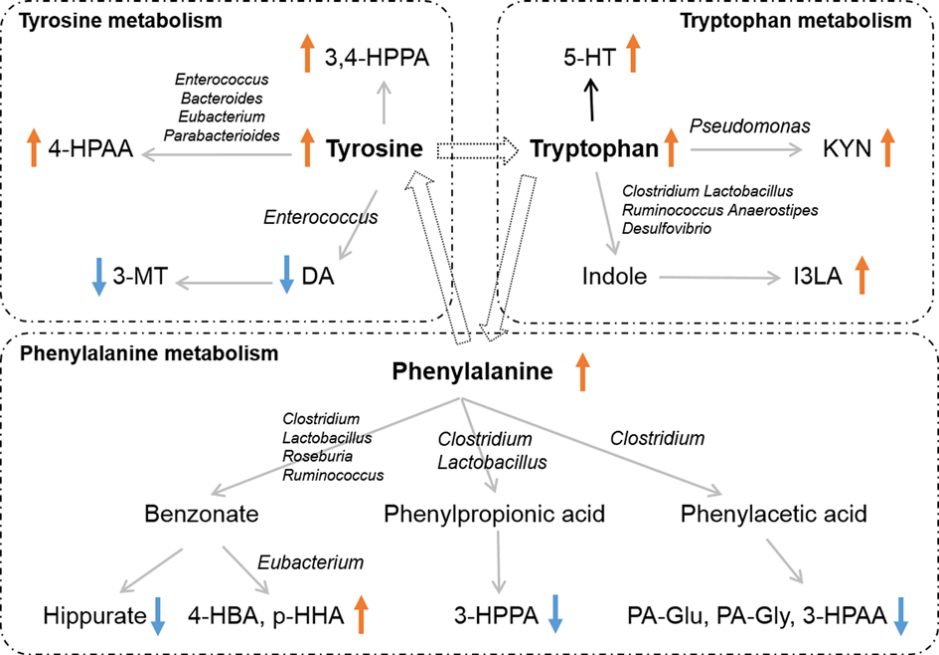
Summarized alteration in gut microbiota and the disturbance of AAAs in mice treated with long-term antibiotics

The increased levels of urinary tyrosine, 3,4-HPPA, and 4-HPAA, and the decreased levels of DA and 3-MT revealed the effect of gut microbial disorder on tyrosine metabolism, which indicated the long-term antibiotic intervention stimulated conversion from tyrosine into 3,4-HPPA and 4-HPAA, and inhibited the synthesis of DA and 3-MT (Figure 4). DA is a naturally occurring catecholamine of neurotransmitters participating in various physiological functions [19]. Its deficiency can result in depression [20], anxiety [21], fatigue [22], and memory impairment [23], which have been reported in previous antibiotics-treated animal models [24] and patients [25]. This implied the possible mechanisms for neurological defects or symptoms after long-term antibiotics treatment. Maini et al. found that *Enterococcus* can help tyrosine decarboxylase (TDC) to degrade tyrosine into DA [26]. Here we found *Enterococcus is* not only shows a negative correlation with DA and 3-MT significantly but also shows a positive correlation with 4-HPAA (Figure 3). It may be because TDC not only participates in the transition from tyrosine into DA and 3-MT but also involves the conversion from tyrosine into 4-HPAA. Besides, the *Bacteroides, Clostridium, Eubacterium*, and *Parabacterioides* also participate in the conversion of tyrosine into 4-HPAA [27], which consisted of the positive correlation between *Enterococcus, Bacteroides, Eubacterium, Parabacterioides* and 4-HPAA in our results.

The conversion from phenylalanine into 4-HBA and p-HHA, together with the suppressed levels in phenylacetic acid (PAA), phenyl propionic acid (PPA) and benzoate (BA) pathway that indicated the involvement of the phenylalanine pathway after antibiotics treatment (Figure 4). Previous studies have shown that phenylalanine enrichment mediated by gut microbiota can promote immune cells to infiltrate the brain, leading to pathological neuroinflammation [28]. Such inflammation with differentiation and proliferation of pro-inflammatory T helper 1 cells were also found in broad-spectrum antibiotics induced model [28,29]. The up-regulated phenylalanine in our results may imply the inflammatory state and impaired immune system in the long-term antibiotics model. It is known that the *Clostridium, Bacteroides*, and *Eubacterium* help phenylalanine metabolize into PAA through the oxidation pathway, so that the PAA could conjugate with glycine or glutamine to form PA-Gly and PA-Gln [12,27]. The plasma PA-Gln is a molecular that could involve in the enhancement of platelet thrombotic potential via adrenergic receptors, which associate with the incident risk of heart attack, stroke, and death [30]. The decreased urinary PA-Gln here may imply the possible risk of cardiovascular disease in long-term antibiotics treatment. Besides, the phenylalanine also could convert into PPA and 3-HPPA under the existence of *Clostridium, Lactobacillus*, and *Bacteroides* [10,31]. The urinary metabolite 3-HPPA/PA-Gln/PA-Gly were also found to highly correlate with *Clostridium/Bacteroides* in our results. Except for this, the phenylalanine also could metabolize into phenolic, benzoyl, and phenyl derivatives such as BA, HA, and 4-HBA with help of *Clostridium, Roseburia, Ruminococcus, Eubacterium*, and *Lactobacillus* [27,31]. The *Clostridium, Roseburia, Ruminococcus*, and *Lactobacillus* were positively correlated with decreased HA in our results. In contrast, the *Eubacterium* was positively correlated with the increased levels of 4-HBA and p-HHA (Figure 4). This suggests that the decreased abundance of *Clostridium, Roseburia, Ruminococcus, Lactobacillus*, and increased abundance of *Eubacterium* affected the catabolism from phenylalanine into PAA, BA, 4-HBA, and PPA after the long-term antibiotics intervention.

Tryptophan is another essential AAA that can mediate the interaction between the host and its gut microorganisms by three major pathways: KYN pathway, 5-HT pathway, and indole pathway [32]. The KYN pathway is the main pathway of tryptophan metabolism with the aid of *Pseudomonas* [33]. Agus et al. found the release of inflammatory cytokines can active IDO (Indoleamine 2,3-dioxygenase 1), a rate-limiting enzyme for transforming tryptophan into KYN in the gut [32]. Here the up-regulation of KYN and IDO (KYN/tryptophan ratio [33,34]) in serum and cecum may suggest stimulated inflammatory status [35] and immune reaction [36] in long-term antibiotics treatment. Zarrinpar et al. proved that the up-regulation of pro-inflammatory cytokines IL-6 and IL-12 in a combined treatment of ampicillin and vancomycin mice model [37]. Knoop et al. found the increased inflammatory cytokines CXCL1, IL-17, and IFN-γ in single dose of a combination of four antibiotic treatments [38], which confirmed the antibiotics promoted inflammation through the bacteria translocation. Besides, under the condition of diminished intestinal microorganisms, the indole-related metabolites produced by tryptophan should be suppressed, along with the up-regulated KYN and 5-HT in the antibiotics mice. Several bacterial species have been reported to help transform tryptophan into indole and its derivatives such as *Clostridium, Lactobacillus, Ruminococcus, Anaerostipes, Desulfovibrio, Bacteroides, Eubacterium, Enterococcus*, and *Parabacteroides* [39]. However, because of the difficulty in coverage of detecting all the indole and its derivatives in our results, we did not find the direct correlation between indole and its derivatives with bacterial species. But the negative correlation between metabolites in the KYN/5-HT pathway and the *Clostridium, Lactobacillus, Ruminococcus, Anaerostipes, Desulfovibrio* support it indirectly. It was known that indole and its derivatives are endogenous ligands of AHR [40], which mediates a variety of physiological functions involving immunity and intestinal homeostasis [41,42]. Based on the decreased levels of metabolites in the indole pathway, mice with altered intestinal flora lack sufficient AHR ligands and further aggravate the intestinal inflammation [36]. The decreased expression of AHR in the colon tissue after antibiotic treatment further confirmed such hypothesis.

In conclusion, the long-term antibiotic treatment significantly changed the composition of the gut microbiota and destroyed the homeostasis in the intestinal metabolism. The host–gut microbiota disturbance manifested as accumulated AAA and re-distribution of their down-streaming metabolites. The correlation between gut microbiota and AAA metabolites could provide essential information for selecting the functional bacteria, which could be helpful in optimizing therapeutic strategies for long-term antibiotics induced dysbiosis in the near future. Our results also revealed the AAA profiles could be an indicator for exploring interactions between host and gut microbiota.

## Data Availability

The data used to support the findings of the present study are available from the corresponding author upon request.

## Abbreviations

3,4-HPPA: 3-(3,4-dihydroxy phenyl) propionic acid
3-HPAA: 3-hydroxyphenyl acetic acid
3-HPPA: 3-(3-hydroxyphenyl) propanoic acid
3-MT: 3-methoxytyramine
4-HBA: 4-hydroxybenzoic acid
4-HPAA: 4-hydroxyphenyl acetic acid
5-HT: serotonin
AAA: aromatic amino acid
AHR: aryl hydrocarbon receptor
BA: benzoate
cDNA: complementary DNA
DA: dopamine
F/B: Firmicutes/Bacteroidetes
FDR: false discovery rate
HA: hippuric acid
I3LA: indole-3-lactic acid
IDO: indoleamine 2,3-dioxygenase 1
IS: internal standard
KYN: kynurenine
LC-MS/MS: liquid chromatography-tandem mass spectrometry
OTU: operational taxonomic unit
*p*-HHA: *p*-hydroxyhippuric acid
PA-Gln: phenylacetyl glutamine
PA-Gly: phenylacetyl glycine
PAA: phenylacetic acid
PPA: phenyl propionic acid
TDC: tyrosine decarboxylase
